# CONTRIBUTIONS TO AGING RESEARCH BY DOCTORATES IN GERONTOLOGY AND GERIATRICS: A PORTUGUESE CASE STUDY

**DOI:** 10.1093/geroni/igad104.2112

**Published:** 2023-12-21

**Authors:** Oscar Ribeiro, Soraia Teles, Constanca Paul

**Affiliations:** University of Aveiro, Aveiro, Aveiro, Portugal; University of Porto, Institute of Biomedical Sciences Abel Salazar, Porto, Porto, Portugal; ICBAS, Portugal, Porto, Porto, Portugal

## Abstract

Portugal is home to one of the world’s oldest populations, ranking in the top five OECD countries with the highest share of people aged 65 and over. The PhD in Gerontology and Geriatrics (PDGG) is the first and one-of-its-kind response in Portugal to the need of advanced training in the field. This work describes the contributions of PDGG doctorates from 2009 to 2022. Electronic/paper-based academic records were retrieved to characterize the doctorates’ entry profiles. Documentary analysis of dissertations was performed to describe their research. Related publications and its impact were identified from searches on academic databases/engines. Doctorates (N=36; 88.9% female) were aged between 23 and 54 (M 33.0). The background is in health and social sciences, with more representation from nurses (30.6%) and psychologists (22.2%). Approximately thirty percent (n=10) had a Masters in Gerontology, and all were professionally experienced with older adults (1-29 years). Doctoral research topics included cognitive impairment and mental health, healthcare/social services/interventions; intergenerationally/family relations/informal care; frailty; nutrition and physical activity, minority populations, elder abuse and spirituality. The research of the 36 doctorates involved 12374 participants (9499 older persons). Outputs comprised 153 publications in scientific journals, receiving 1640 citations, 87782 usages (e.g., views, downloads), 9538 captures (e.g., exports) and 541 in social media or news articles (PlumX metrics). Other contributes included 8 original intervention programs, 3 new and 11 translated/culturally-adapted assessment scales. This case study shows the intergenerational and lifelong learning environment of the PDGG, and its many contributions to the the aging field.

